# Gaussian Curvature Effects on Graphene Quantum Dots

**DOI:** 10.3390/nano13010095

**Published:** 2022-12-25

**Authors:** Sergio de-la-Huerta-Sainz, Angel Ballesteros, Nicolás A. Cordero

**Affiliations:** 1 Physics Department, Universidad de Burgos, E-09001 Burgos, Spain; 2 International Research Center in Critical Raw Materials for Advanced Industrial Technologies (ICCRAM), Unversidad de Burgos, E-09001 Burgos, Spain; 3 Institute Carlos I for Theoretical and Computational Physics (IC1), E-18016 Granada, Spain

**Keywords:** graphene, Gaussian curvature, quantum revival, DFT, pseudo-magnetic field, phase transition

## Abstract

In the last few years, much attention has been paid to the exotic properties that graphene nanostructures exhibit, especially those emerging upon deforming the material. Here we present a study of the mechanical and electronic properties of bent hexagonal graphene quantum dots employing density functional theory. We explore three different kinds of surfaces with Gaussian curvature exhibiting different shapes—spherical, cylindrical, and one-sheet hyperboloid—used to bend the material, and several boundary conditions regarding what atoms are forced to lay on the chosen surface. In each case, we study the curvature energy and two quantum regeneration times (classic and revival) for different values of the curvature radius. A strong correlation between Gaussian curvature and these regeneration times is found, and a special divergence is observed for the revival time for the hyperboloid case, probably related to the pseudo-magnetic field generated by this curvature being capable of causing a phase transition.

## 1. Introduction

While early stages of graphene research were centered on its theoretical aspects [1,2,3,4], after the successful isolation of a single sheet of graphitic material by Geim and Novoselov in 2004 [5], there has been a long trend of advancements populated with experimental confirmation of predicted properties, the discovery of new and exotic phenomena, and improvements to the synthesis methods for this material. The ever-growing list of potential applications of graphene spreads across many fields due to its outstanding properties and exotic behaviors, such as engineering [6,7,8], medicine [9,10,11,12], sensor fabrication [13,14,15,16], catalysis [17,18,19,20], energy storage and management [21,22,23], and flexible and high-performance electronic devices [24,25,26,27,28,29]. Graphene nanostructures have had even greater potential since the discovery of superconductivity in bilayer graphene [30,31,32].

Quantum revival—the periodic regeneration of the initial state of a time-dependent quantum system—on the other hand, is still a subject mainly studied from a theoretical point of view, though its experimental realization is possible and opens up interesting research directions for information transmission and the fabrication of quantum devices. For instance, quantum revivals can be used to measure fidelity in quantum information technology, and this measurement is crucial in order to understand the effects of decoherence, dissipation, and imperfections in quantum information devices [33]. In addition, revivals have been proposed as a method for transporting information with high efficiency or generating entanglement [34]. On a wider view, the temporal evolution of a system has been quite useful for real-time screening of high-speed phenomena, such as chemical reactions [35,36,37,38].

Thus, we aim to study how different factors, such as shape and Gaussian curvature, change the behavior of a graphene-based system through the simulation of its quantum revivals. This is especially relevant, since graphene, which is commonly conceived as a perfectly flat and pristine sheet of carbon atoms, has naturally a far more complex structure, with ripples, wrinkles, and many other deviations from its ideal flatness [39,40,41,42,43,44,45], corroborating theoretical predictions done many years before its isolation [46,47,48]. While the true origins of these deviations from flatness are still up for debate, their influences on the material properties, such as charge transport, allow for fine-tuning of its behavior [49,50,51,52].

Other carbon nanostructures can be classified based on their Gaussian curvature: carbon nanotubes [53,54,55,56] and nanocones [57,58,59,60,61]—and ideal graphene—have null Gaussian curvature, as the material stays flat in at least one of its principal directions. Others, such as fullerenes, form closed structures having positive curvatures. The case of negative Gaussian curvature has been more elusive, but it has been found in schwarzites [62,63] as open or even periodic structures in which each point resembles a saddle.

In fact, there have been recent advancements on the synthesis and design of carbon nanostructures and polycyclic aromatic systems with tunable curvature—via incorporation of pentagons or heptagons [64,65,66]—or even modeling of hybrid systems between flat and curved structures [67], with important applications for batteries development and engineering. Negatively curved graphene has also been used as an analog for gravitational systems [68,69], allowing direct observation of exotic behaviors in a much more approachable fashion.

We recently presented results on spherically deformed graphene quantum dots [70]. Those nanostructures have positive Gaussian curvature. We expand our study in this article to negative and null Gaussian curvatures, presenting a comparative study of curvature effects in energy and electronic structure obtained using density functional theory, on graphene quantum dots with various Gaussian curvature values.

## 2. Materials and Methods

From the many possibilities available for the theoretical study of graphene, each of them with its own advantages, reliability, and range of application, we chose the procedure described in our previous work [70] and used density functional theory (DFT) [71,72,73] as the main tool for simulating the properties of graphene quantum dots through use of the the Gaussian 16 [74] package. For the exchange-correlation functional, local density approximation (LDA) [75,76] was used, in virtue of its better performance for graphitic systems and higher calculation speed compared with general gradient approximations (GGAs) or hybrid functionals such as B3LYP [77,78,79,80,81], and because it has been successfully used to analyze interactions in carbon nanostructures [82,83,84,85,86,87]. The basis set was 6-31G** [88], with d-type and p-type functions as polarization aids for a better description of the chemical bond.

**Figure 1:**
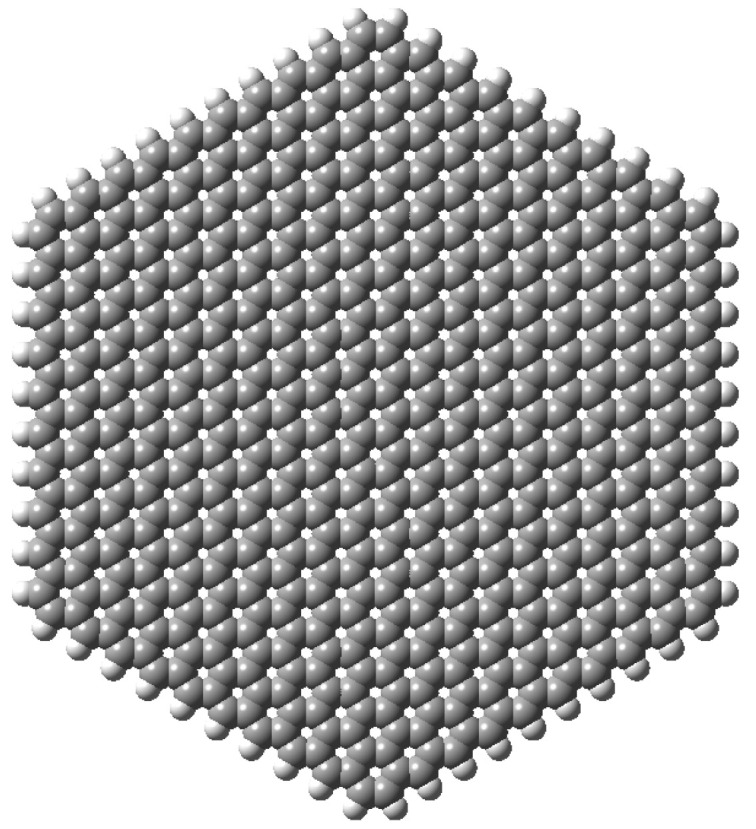
Hexagonal, flat graphene quantum dot used as a starting point for deformation. Image generated with Gaussview 6 [91].

We studied a hexagonal graphene quantum dot with 10 carbon atoms on each edge and hydrogen passivated resulting in a $C_{600}H_{60}$ molecular formula (see Figure 1). All edges were of the zig-zag type to avoid the appearance of other phenomena, such as asymmetric or unbalanced magnetic states [89,90] or significant repulsive interactions between passivating atoms. This system, with a distance between vertices of 46.4 Å, allowed us to achieve a compromise between the experimental size of graphene natural corrugation [39,41] and computational cost.

The focus of this paper is on the effects of different Gaussian curvature values (null, positive and negative) for the material, and for that we employed a family of surfaces for bending the dot. All these surfaces have a common expression that can be written in Cartesian coordinates as:

(1) $$z=\sqrt{R^{2}-ax^{2}-by^{2}}\;.$$

**Figure 2:**
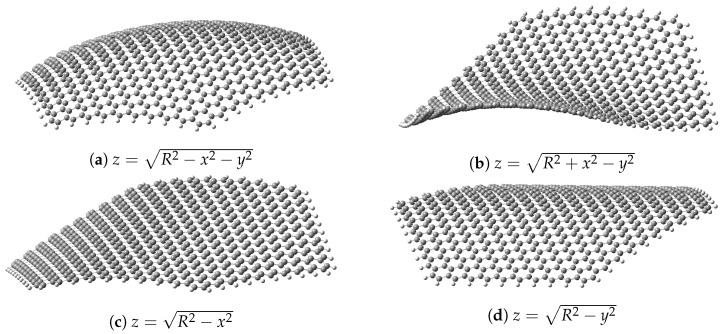
The four different geometries considered in this study for the graphene dot with R=50 Å and their respective equations: (**a**) sphere; (**b**) one-sheet hyperboloid; (**c**) x-cylinder; (**d**); y-cylinder. Images generated with GaussView 6 [91].

From Equation (1), different kinds of Gaussian curvature can be obtained: (i) positive, for the sphere (a=b=1); (ii) zero, for the cylinder (either *a* or *b* being 1, the other being 0); and (iii) negative, for the one-sheet hyperboloid—referred to simply as hyperboloid from now on (either *a* or *b* being 1, the other being −1). Although the two possible cylinders are equivalent for a square dot, we simulated both as separate surfaces, as the hexagonal dot’s final geometry is different; we call them cylinders x and y regarding which component has a non-zero coefficient. For the hyperboloid, however, exchanging these coefficient values only provides a flipped (and thus equivalent) structure, so only one case was considered. These surfaces can be seen in Figure 2.

**Figure 3:**
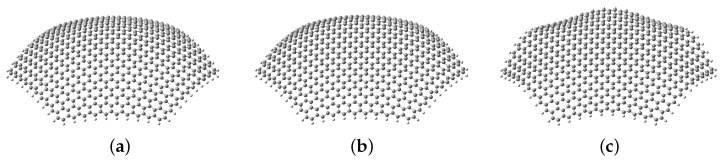
Boundary conditions’ effects on the optimized geometries of an initially spherical quantum dot with R=40 Å. Images generated with Gaussview 6 [91]. (**a**) Fixed surface; (**b**) fixed edges; (**c**) fixed vertices.

The boundary conditions of the dot were a second factor in this study because they are important for the experimental realization of these systems. The ideal case, where all atoms are confined to the initial surface, would be impractical to reproduce at this scale; therefore, we consider two additional possibilities more feasible for an experimental setup. We used three cases for each surface regarding what atoms were restrained: (i) all 600 carbon atoms must remain on the surface; (ii) only the 60 edge carbon atoms are fixed; (iii) only the 12 vertex carbon atoms are fixed. Figure 3 shows these possibilities for the spherical case with R=40 Å. This set of decreasing restrictions allows the curved dot to relax further in an attempt to recover far from the edges its initial and optimal flat shape.

## 3. Results and Discussion

In this study, we have focused on the analysis of curvature energy and quantum regeneration times for our hexagonal dot as it is deformed according to the different surfaces described and considering each set of boundary conditions. The parameter $1/R^{2}$ is used in all graphics as a measure of the curvature of the dot. While this is only true for the perfectly spherical case (having the Gaussian curvature as zero for the cylinder, and negative, non-constant for the hyperboloid), we use it for comparative purposes for quantification of the deformation.

### 3.1. Curvature Energy

Curvature energy, calculated as the difference between the energy of a given dot and that of the flat one, has been calculated for all available cases and plotted against the $1/R^{2}$ parameter. This enables a quick inspection of the stability of the system from a mechanical point of view, allowing us to check what surfaces and boundary conditions lead to more stable systems.

**Figure 4:**
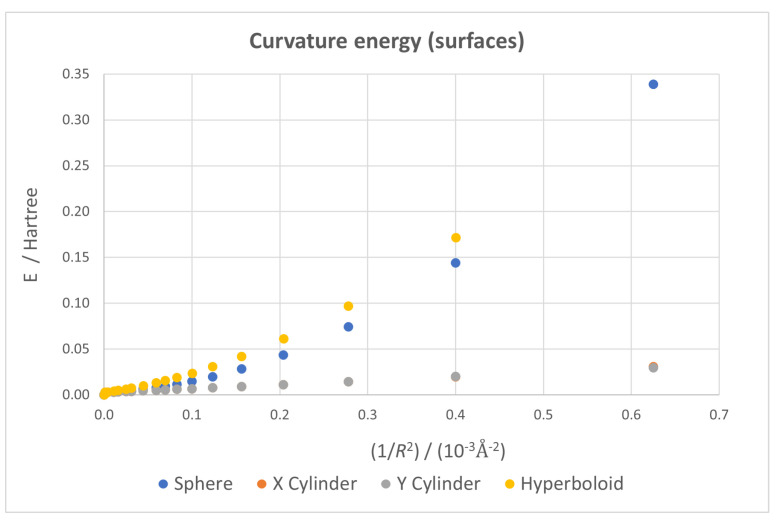
Curvature energy vs. $1/R^{2}$ for all four ideal geometries—all atoms forced to lay on the surface—with the flat dot taken as energy origin. Both cylindrical cases give almost identical energies.

In order to analyze the effects of the two factors considered in this study, we first consider the type of surface used, plotting the curvature energies for all the ideal geometries (that is, with all carbon atoms lying on the surface) in Figure 4. As expected, curvature energy for all surfaces increases as curvature does; higher values exist for hyperboloidal and spherical surfaces than for cylindrical ones, which give nearly identical results (proving the near equivalence of the two cylindrical structures from a mechanical point of view). These results are consistent with the fact that for spherical and hyperboloidal surfaces, the deformation is applied along two spatial axes instead of only one, as in the cylinder. The slightly higher instability for the hyperboloid case is derived from the inherent general stretching of the structure that forces a larger deviation from the sp${}^{2}$ hybridization of planar graphene than in the spherical case.

**Figure 5:**
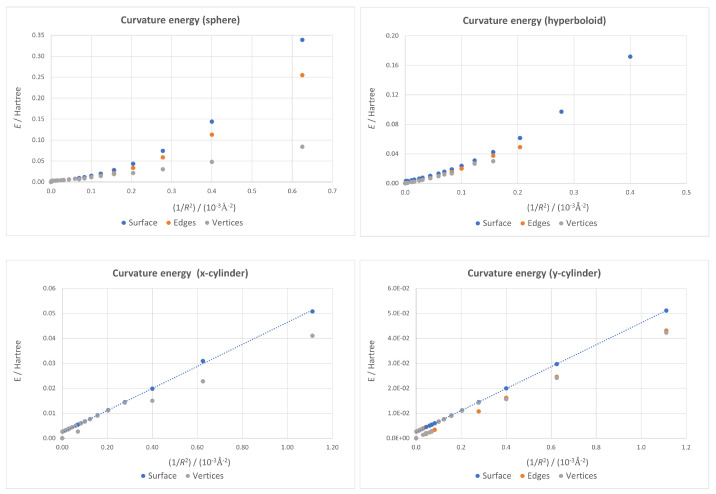
Curvature energy vs. $1/R^{2}$ plots for all geometries, with the flat dot taken as energy origin.

The effects of the boundary conditions applied to each dot are plotted in Figure 5 for each surface so that relative changes in the general behavior can be easily observed. For all four surfaces considered, the cases where only the vertices were fixed are the most stable ones, as expected, while the ideal geometries represent a nearly optimal structure only for small curvature values. In the hyperboloid case, the deviation from the ideal surface starts from very small curvatures, and the energy gain when relaxing boundary conditions is bigger. Nevertheless, it was not possible to get results for high values of $1/R^{2}$. This is probably due to the fact that a big deviation from the planar case with opposite signs in different directions leads to the breaking of the nanostructure. A dynamical (for instance, molecular dynamics) calculation would be necessary to confirm this hypothesis.

While the fixed-surface cylindrical plots are essentially straight lines, as the least-squares linear fits plotted in Figure 5 prove—showing the linear dependence on $1/R^{2}$ characteristic of the continuum model applied to a nanotube [92]—the spherical and hyperboloidal ones are not. In our previous work [70], it was shown how this discrepancy with the continuum model could be connected in the spherical case to the small position changes derived from the use of quantum mechanics in the optimization instead of a classical force field. However, these new results prove that the continuum model is indeed valid for the cylindrical cases—that are curved surfaces—even when quantum mechanics is used to determine geometries, suggesting there are other important contributions besides the theory used for geometry optimization. It seems that structures with non-zero Gaussian curvature suffer additional strain that the continuum model cannot take into account.

Since the continuum model is valid for the cylindrical case, it is possible to calculate the bending modulus of this graphene quantum dot by making use of the least-squares fits depicted in the lower two panels of Figure 5. The bending energy of a nanotube of radius *R* can be written as [93,94]:

(2) $$E=\frac{C_{b}}{2R^{2}},$$

with $C_{b}$ being the bending modulus (also known as flexural rigidity). Our graphene quantum dot is not a carbon nanotube, but, taking into account that all atoms on its borders are passivated, there are no dangling bonds, and the nanostructure can be considered as a piece of the wall of a nanotube. Looking at Equation (2), $C_{b}$ is just twice the slope of the linear fit. For the x-cylinder, this calculation leads to $C_{b}=4.00$ eV Å${}^{2}$ per C atom, and for the y-cylinder, it yields $C_{b}=3.99$ eV Å${}^{2}$. Both results are nearly identical, in spite of the fact that the x-cylinder could be considered as a piece of a zig-zag nanotube, while the y-cylinder would correspond to a piece of an armchair tube. The bending modulus of carbon nanotubes being independent of the bending direction is a well-known fact [95] and a consequence of the hexagonal symmetry of the graphene lattice that makes this material isotropic in the linear elastic regime [96]. Our results are in excellent agreement with those obtained for the bending modulus per C atom by Kürti et al. (3.9±0.1 eV Å${}^{2}$) [97], Sánchez-Portal et al. (4.00 eV Å${}^{2}$ for armchair tubes) [98] and Kudin et al. (3.9 eV Å${}^{2}$) [93].

An additional piece of information we can extract from the plots corresponding to both cylindrical cases in Figure 5 is about the accuracy of our calculations. For very low values of $1/R^{2}$ (below $10^{-4}\;Å{}^{-2}$), there are some jumps when atoms are allowed to relax outside the ideal cylindrical surface. Sometimes there is a gain in energy, but other times there is no gain, showing that the geometry had not fully relaxed because the code had not detected the additional stabilization due to breaking the exact cylindrical shape. These jumps are about 2 milliHartree, and this value can be taken as an estimation of the accuracy of the method.

### 3.2. Regeneration Times

The study of quantum revival phenomena was carried out using the eigenvalue spectra obtained for each dot, performing an analysis of the electronic properties of our system by means of a homemade code built within the Mathematica environment [99]. In order to calculate these revival phenomena, we define, following Robinett [100], the initial state of a time-independent wavepacket as a linear combination of eigenstates $|u_{n}\rangle$ with weights $a_{n}$:

(3) $$|\Psi (0)\rangle =\sum_{n=0}^{\infty }a_{n}|u_{n}\rangle \;,$$

with its temporal evolution having the following expression:

(4) $$|\Psi (t)\rangle =\sum_{n=0}^{\infty }a_{n}|u_{n}\rangle e^{-\frac{i}{\hbar }E_{n}t}\;,$$

where $E_{n}$ is the the eigenenergy of $|u_{n}\rangle$.

Since we are using the energy spectrum calculated with DFT to build the wavepacket, we can take one single level as a central point and perform a Taylor expansion around it to get an analytical expression for the spectrum:

(5) $$E_{n}=E_{n_{0}}+E_{n_{0}}^{{}^{\prime }}\left(n-n_{0}\right)+\frac{1}{2!}E_{n_{0}}^{{}^{\prime \prime }}{\left(n-n_{0}\right)}^{2}+\frac{1}{3!}E_{n_{0}}^{{}^{\prime \prime \prime }}{\left(n-n_{0}\right)}^{3}+\ldots .$$

After substituting this expansion into Equation (4), the final temporal evolution shows several terms inside the exponential, each of them corresponding to one time scale and giving rise to different regeneration times (classical, $T_{\mathrm{Cl}}$; revival, TRe; superrevival, $T_{\mathrm{Sup}}$; …):

(6) $$|\Psi (t)\rangle =\sum_{n=0}^{\infty }a_{n}|u_{n}\rangle e^{-\frac{i}{\hbar }\left[E_{n_{0}}+E_{n_{0}}^{{}^{\prime }}\left(n-n_{0}\right)+\frac{1}{2!}E_{n_{0}}^{{}^{\prime \prime }}{\left(n-n_{0}\right)}^{2}+\frac{1}{3!}E_{n_{0}}^{{}^{\prime \prime \prime }}{\left(n-n_{0}\right)}^{3}+\ldots \right]t}\;,$$

(7) $$T_{\mathrm{Cl}}=\frac{2\pi \hbar }{|E_{n_{0}}^{{}^{\prime }}|}\;,$$

(8) TRe=2πħ|En0″|/2!,

(9) $$T_{\mathrm{Sup}}=\frac{2\pi \hbar }{|E_{n_{0}}^{{}^{\prime \prime \prime }}|/3!}\;.$$

As for the wavepacket itself, the coefficients $a_{n}$ will follow a Gaussian distribution,

(10) $$a_{n}=\frac{1}{\sigma \sqrt{\pi }}e^{\frac{-{\left(n-n_{0}\right)}^{2}}{2\sigma^{2}}}$$

centered around the fifth unoccupied orbital (LUMO+4), thereby having a value $n_{0}=5$, and a width σ=0.7, ensuring a small collection of five states with a significant contribution ($a_{n}>0.001$).

Temporal evolution was studied by means of the squared modulus of the autocorrelation function, ${\left|A\left(t\right)\right|}^{2}$, defined as the overlap of the the wavepacket after an arbitrary time *t* and its initial state:

(11) $${\left|A\left(t\right)\right|}^{2}={\left|\langle \Psi (0)|\Psi (t)\rangle \right|}^{2}\;.$$

**Figure 6:**
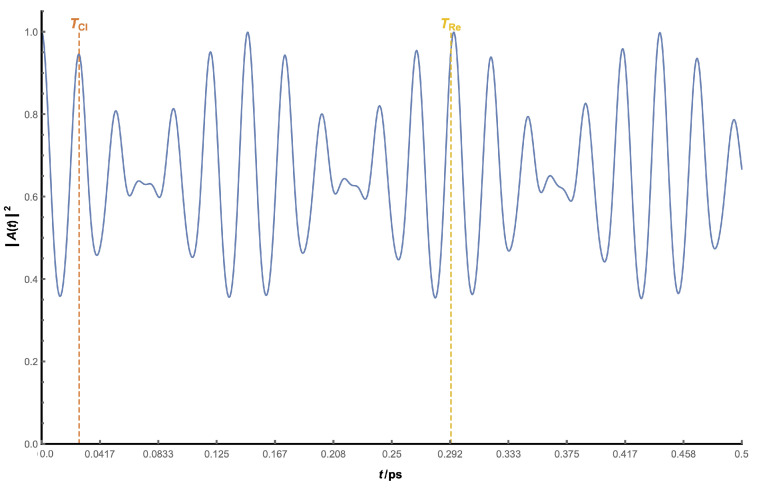
View of ${\left|A\left(t\right)\right|}^{2}$ as a function of *t* for a spherical dot with R=100 Å. Analytical values of both regeneration times are shown with dotted lines (orange for classical time, yellow for revival time).

Figure 6 shows the plot corresponding to a typical example in which the oscillatory patterns of ${\left|A\left(t\right)\right|}^{2}$ are evident. The periodicities at different time scales correspond to different regeneration times, with classic time $T_{\mathrm{Cl}}$ being the high-frequency one, and revival time TRe the low-frequency one. While revival times of higher order (such as $T_{\mathrm{Sup}}$) are theoretically possible, none beyond TRe could be observed in any case due to the interference among different regeneration times.

Obtaining the values of these two regeneration times is an easy task: $T_{\mathrm{Cl}}$ corresponds to the first maximum of ${\left|A\left(t\right)\right|}^{2}$, and TRe comes from the first maximum of the enveloping curve, which can be calculated using the local maxima of ${\left|A\left(t\right)\right|}^{2}$. There can be some difficulties in their determination, however, if TRe is not much larger than $T_{\mathrm{Cl}}$. In this case, interference between those times can occur, making the visual observation of both, especially $T_{\mathrm{Cl}}$, harder. For this reason, the analytical expressions of both times, derived from the Taylor expansion described, have been used as an alternative method and aid in its determination. We have, then, analytical (from Taylor expansion) and numerical (from temporal evolution) values for each time. A parabolic curve, fitted to the three central levels of the wavepacket, has been used as the fitting function for the eigenvalue spectrum in order to calculate derivatives.

In a similar fashion to the energy analysis, the results of this section are presented in two steps: first a study of the effects of the kind of surface, and second, an individual view of each of them upon relaxing boundary conditions.

#### 3.2.1. Classical Time

**Figure 7:**
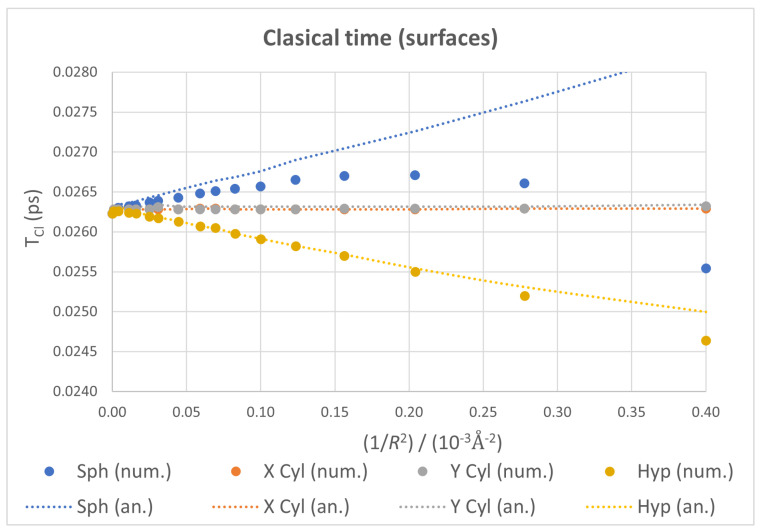
Classical time plots as functions of $1/R^{2}$ for all four ideal geometries, with numerical values as markers and analytical ones as dotted lines. Both cylinders show near perfect coincidence.

The plots for $T_{\mathrm{Cl}}$ against $1/R^{2}$ for all four surfaces considered and all carbon atoms on the dots confined to them are depicted in Figure 7, showing the numerical values as points and the analytical ones as dotted lines just for clarity. For the cylindrical cases, not only do both surfaces give almost identical values—in a similar fashion to what happened with the curvature energy—but $T_{\mathrm{Cl}}$ also remains nearly constant for the whole range of *R* studied. In contrast, the spherical and hyperboloidal cases exhibit opposite behaviors: $T_{\mathrm{Cl}}$ increases with curvature in the former and decreases in the latter. Considering the inverse relation between $T_{\mathrm{Cl}}$ and the first derivative of the spectrum, these results reflect that energy levels get closer as the curvature of the sphere increases, get sparser for the hyperboloid and remain almost unchanged for the cylinder. This group of opposite tendencies and constant behavior aligns with the signs of the Gaussian curvature for the corresponding surfaces.

There is a strong deviation in numerical $T_{\mathrm{Cl}}$ from its analytical counterpart for the sphere at higher values of $1/R^{2}$. This is due, as we commented earlier, to the interference between classical and revival times. As they approach each other, the enveloping curve shifts more the position of the first maximum, distorting the numerical value of $T_{\mathrm{Cl}}$. Since the analytical approach considers only the local shape of the spectrum, this interference cannot be taken into account, and the corresponding plot is nearly a straight line with a slope opposite to that in the hyperboloid case.

**Figure 8:**
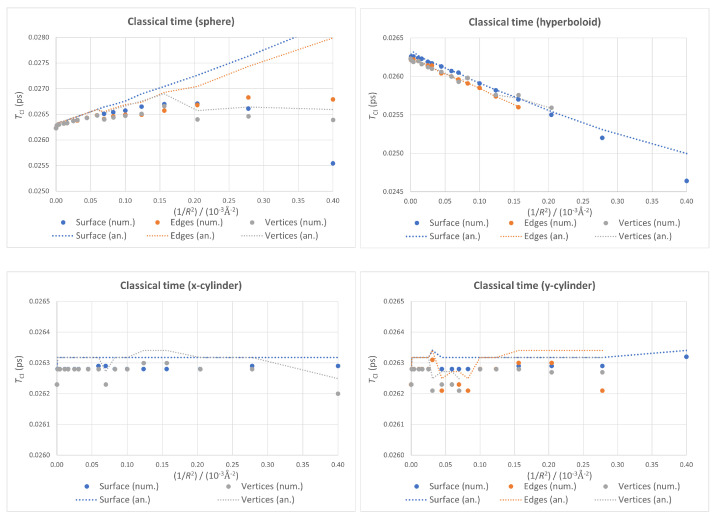
Classical time as a function of $1/R^{2}$ for different boundary conditions within each geometry, with numerical values as points and analytical ones as dotted lines.

When boundary conditions are relaxed (see Figure 8), a similar phenomenon to the one observed for the energy can be seen. While fixed-surface quantum dots give smooth plots with monotonic trends, the changes in the optimal geometry for the other two sets of conditions introduce breaking points into the values of $T_{\mathrm{Cl}}$, resulting in fragmented plots in which the overall trend is otherwise conserved.

Again, the plots corresponding to both cylindrical cases in Figure 8 can be used to estimate the accuracy of our calculations. The jumps for values of $1/R^{2}$ below $10^{-4}\;Å{}^{-2}$ can be taken as an indication of the accuracy of the classical regeneration times we calculated: Around 1 fs.

#### 3.2.2. Revival Time

**Figure 9:**
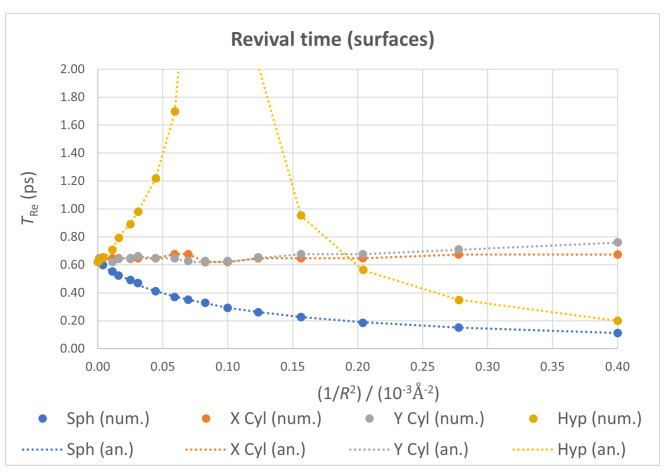
Revival time as a function of $1/R^{2}$ for all four ideal geometries, with numerical values as points and analytical ones as dotted lines.

A comparison of TRe for the different kinds of ideal surfaces considered can be seen in Figure 9. While revival time shows again a nearly constant value for the two cylindrical cases, it decreases nonlinearly for the spherical geometry, and exhibits clearly divergent behavior in the hyperboloidal case for $1/R^{2}≃10^{-4}\;Å{}^{-2}$. Again, this contrast of trends has a one-to-one correspondence with the sign of the Gaussian curvature of each surface.

TRe being inversely proportional to the second derivative of the spectrum gives information about its linearity and relative separation between consecutive levels. The divergence observed in the hyperboloid suggests an ideally infinite value of TRe, due to a null second derivative caused by the levels being equally spaced in energy for a special value of *R*.

In our previous work, we suggested this kind of behavior could be connected to the generation of pseudo-magnetic fields on the material, as had been found for flat systems with electric fields applied, possibly as a sign of a phase transition [101,102,103]. These pseudo-magnetic fields have been measured in graphene upon applying a periodic, negative curvature [104,105], allowing us to link both findings with our results and hypothesize that negative Gaussian curvature creates pseudo-magnetic fields on the material that could cause a phase transition, observable through a divergence in the quantum revival time. Further tests with other negative curvature surfaces are needed to confirm this hypothesis.

The divergence found for the hyperboloid case looks similar to the divergence found in our previous work [70] for the fixed-surface spherical case with atom displacement from the configuration corresponding to the quantum-mechanical energy minimum, but it has nothing to do with it. There, the divergence was an effect of using a non-self-consistent calculation but a perturbative one. Here, it is the result of a fully self-consistent procedure. Therefore, while the possible phase transition was then an effect of a small distortion of the equilibrium geometry, it is now just a bending consequence present in the true minimum energy configuration.

**Figure 10:**
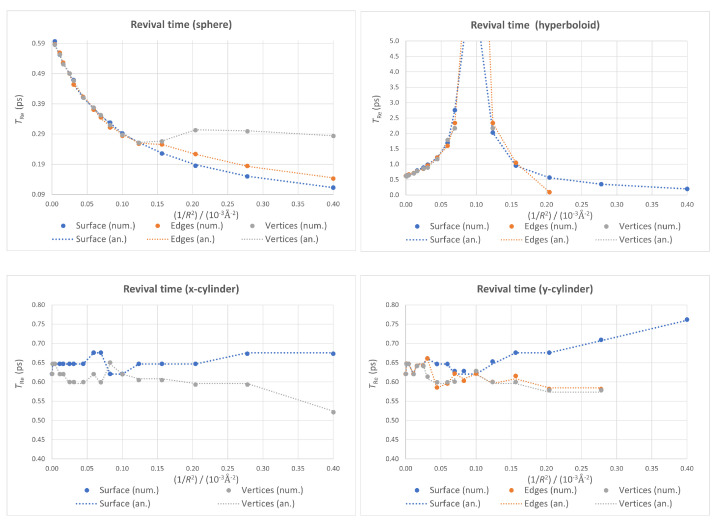
Revival time as a function of $1/R^{2}$ for different boundary conditions within each geometry, with the numerical values as markers and the analytical ones as dotted lines.

The effects of the boundary conditions on TRe are presented in Figure 10. In the spherical case, relaxing these conditions leads to a progressive change from the monotonic decrease with the appearance first of a shoulder (for the fixed-edges case) and then of a local minimum (for the fixed-vertices case).

Once again, the plots corresponding to both cylindrical cases in Figure 10 can be used to make an accuracy estimation. The jumps for very low values of $1/R^{2}$ (below $10^{-4}\;Å{}^{-2}$) are an indication of the accuracy of our revival times: around 0.05 ps. It is therefore not clear if the change in tendency for large values of $1/R^{2}$ (i.e., high bendings) from slightly increasing to slightly decreasing in the two cylindrical cases is real or not.

Finally, in the hyperboloid case, relaxing the boundary conditions does not affect the global behavior of the calculated revival times. The divergence seems to be a robust feature, making it a good candidate for experimental confirmation.

## 4. Conclusions

In a recent work [70], we presented results for mechanical and electronic properties of spherically-deformed graphene quantum dots. Spheres have positive Gaussian curvature. In order to better understand curvature effects on these dots, we expanded our study to hyperboloidal shapes (negative Gaussian curvature) and cylindrical structures (zero Gaussian curvature).

We studied both mechanical (equilibrium configurations and curvature energies) and electronic (quantum regeneration times) properties of curved graphene quantum dots. The results obtained for mechanical properties agree with expected behaviors (curvature energy grows with the deformation of the dot; hyperboloidal deformations are energetically less favorable than cylindrical ones, and these are, in turn, less stable than cylindrical shapes; and within each kind of deformation, lifting constraints translates into lower energies, as the dot is capable of getting closer to a flat structure). In the cylindrical case, the bending modulus was calculated, and in spite of being non-closed systems, the result agrees with that for carbon nanotubes. This shows that a finite cylindrical graphene dot’s mechanical response to bending is the same as that of infinite carbon nanotubes walls.

Regarding regeneration times, several trends were obtained that link them to Gaussian curvature. When all atoms are forced to lie on the surface, classical times increase with $1/R^{2}$ for spherical shapes (positive curvature), decrease with $1/R^{2}$ for hyperboloidal flakes (negative curvature) and remain nearly constant for cylindrical surfaces (zero curvature). Revival times decrease with $1/R^{2}$ for spherical shapes; increase with $1/R^{2}$ until they diverge and then decrease for hyperboloidal flakes; and remain nearly constant for cylindrical surfaces. When only peripheral atoms are kept fixed, the general trends do no change, except for the revival time, in the spherical deformation case, where a shoulder appears when only border atoms are fixed, and it changes to a local minimum when only vertex atoms are kept fixed.

The change by several orders of magnitude in the revival time for hyperboloidal systems when $1/R^{2}≃10^{-4}\;Å{}^{2}$ makes them an excellent candidate for experimental confirmation. This kind of divergence has been previously found for graphene rings [101] or graphene flakes in perpendicular magnetic fields [102], but never in the absence of external fields. It has also been found for silicene in a perpendicular electric field [103], and in this case, the reason is a topological phase transition from a topological insulator to a band insulator. It is known that bending graphene creates a pseudo-magnetic field [106]. Therefore, the divergence in the revival time for hyperboloidal systems could be due to a phase transition related to a pseudo-magnetic field created by negative Gaussian curvature in the quantum dot.

## Data Availability

The data presented in this study is contained within the article.

## Abbreviations

The following abbreviations are used in this manuscript:

DFT	Density Functional Theory
GGA	Generalized Gradient Approximation
LDA	Local Density Approximation
LUMO	Lower Unoccupied Molecular Orbital
